# Attitudes among transplant professionals regarding shifting paradigms in eligibility criteria for live kidney donation

**DOI:** 10.1371/journal.pone.0181846

**Published:** 2017-07-21

**Authors:** Jeffrey A. Lafranca, Emerentia Q. W. Spoon, Jacqueline van de Wetering, Jan N. M. IJzermans, Frank J. M. F. Dor

**Affiliations:** 1 Department of Surgery, division of HPB and Transplant Surgery, Erasmus MC, University Medical Center Rotterdam, Rotterdam, The Netherlands; 2 Department of Internal Medicine, division of Nephrology, Erasmus MC, University Medical Center Rotterdam, Rotterdam, The Netherlands; Centre Hospitalier Universitaire Vaudois, SWITZERLAND

## Abstract

**Background:**

The transplant community increasingly accepts extended criteria live kidney donors, however, great (geographical) differences are present in policies regarding the acceptance of these donors, and guidelines do not offer clarity. The aim of this survey was to reveal these differences and to get an insight in both centre policies as well as personal beliefs of transplant professionals.

**Methods:**

An online survey was sent to 1128 ESOT-members. Questions were included about several extended donor criteria; overweight/obesity, older age, vascular multiplicity, minors as donors and comorbidities; hypertension, impaired fasting glucose, kidney stones, malignancies and renal cysts. Comparisons were made between transplant centres of three regions in Europe and between Europe and other countries worldwide.

**Results:**

331 questionnaires were completed by professionals from 55 countries. Significant differences exist between regions in Europe in acceptance of donors with several extended criteria. Median refusal rate for potential live donors is 15%. Furthermore, differences are seen regarding pre-operative work-up, both in specialists who perform screening as in preoperative imaging.

**Conclusions:**

Remarkably, 23.4% of transplant professionals sometimes deviate from their centre policy, resulting in more or less comparable personal beliefs regarding extended criteria. Variety is seen, proving the need for a standardized approach in selection, preferably evidence based.

## Introduction

The increased global incidence and prevalence of diabetes, hypertension, obesity and other risk factors for kidney disease is associated with an increased incidence of end-stage renal disease (ESRD).[1] The golden standard treatment for patients with ESRD is kidney transplantation, but unfortunately, this field is still suffering from the lack of donor organs.[2] Luckily, during the last decades, live kidney donation has proven to successfully expand the donor pool; unfortunately, it still cannot meet the demand for donor kidneys.[2] This, together with the excellent results of live donor kidney transplantation, leads to an increase in the acceptance of live donors with so called ‘extended donor criteria’, i.e., older donors, overweight/obese donors, donor kidneys with vascular multiplicity, donors with comorbidities, women of childbearing age, and even minors as potential donors.[3] In general, live kidney donors are in good health, and therefore it is critical that these potential donors do not become kidney patients themselves. Especially in extended criteria donors, careful pre-operative screening is of the utmost importance. Since the start of live kidney donation programs, donor assessment and surgical aspects have developed impressively, as well as donor management and follow up.[4,5] As a result, a shift has occurred in relative and absolute contraindications for live kidney donation, and more extended criteria donors are deemed suitable to donate.[3,6] Unfortunately, the contra-indications vary greatly between transplant centres worldwide, and even nationally.[7] Transplant professionals have the responsibility to perform individual risk calculations to ensure donor safety. Therefore, the choice to accept a potential donor becomes a rather subjective issue. We previously published a systematic review to reveal the current opinions of available guidelines regarding extended criteria donors and evidence regarding the outcome of these donors.[3] Current guidelines are not very clear regarding these extended criteria. To retrieve more insight in centre policies and opinions/attitudes of transplant professionals on this topic, we have performed an online survey amongst European Society for Organ Transplantation (ESOT) members to reveal potential differences between centre criteria and personal opinions regarding eligibility criteria of live kidney donors, both between continents as between the European centres.

## Methods

### Study population

The European Society for Organ Transplantation (ESOT) is an umbrella organization under which transplant activities are structured and streamlined in Europe and worldwide. ESOT members are dedicated professional volunteers that represent expert knowledge on donation and transplantation. The organization provides an extensive education programme and her members are involved in generating guidelines in the field of transplantation. Unfortunately, no data is available regarding the exact numbers of transplant clinics or transplant professionals in Europe. As we chose to let the respondents fill out the questionnaire anonymously, we could not give insight in the percentages of submissions from specific centres, nor insight if there were more respondents from the same centre.

### Online survey

An online survey was performed using SurveyMonkey (SurveyMonkey Inc., Palo Alto, California, USA). With ESOT-president approval, the questionnaire was sent to all ESOT-members who were profiled in the member database as ‘surgeon’, ‘physician’ or ‘scientists’ and/or selected ‘kidney’ in their profile. On the first page of the survey, participants were obliged to select whether they were either a (transplant) surgeon or a (transplant) nephrologist. All other categories (researcher or other) were then excluded from rest of the questionnaire. Transplant surgeons were asked whether they perform live donor nephrectomies independently; only if the answer was ‘yes’, they could continue the survey. The questionnaire was anonymous, as to retrieve the most honest answers, since deviating from a centres’ policy might be considered ‘out of line’.

The survey consisted of two parts: a centre criteria part, which was presented to both transplant surgeons and nephrologists, and a personal criteria part, which was presented to transplant surgeons for additional specific surgical questions. In total, the survey consisted of 40 questions; 24 centre criteria questions and 16 personal criteria questions. Questions were included about several extended donor criteria, such as overweight/obesity, age limit, vascular multiplicity, minors and women of childbearing age as donors, renal anatomy and co-morbidities like hypertension, impaired fasting glucose and kidney stones. In the personal criteria part, a five-point Likert scale was presented, rating from 1 ‘very unlikely’ to 5 ‘very likely’. Since it is of great importance that a potential donor is carefully assessed regarding anatomy and function of the kidney(s), we have included several questions about pre-operative radiological imaging and functional testing of the kidneys. The questionnaire is presented in S1 Table. Results were divided into the answers of different European centres (Northwest, Mediterranean and East (for exact division see S2 Table)), to compare policies and attitudes within Europe. Also, data is presented to reveal potential differences between continents in the world (S3 Table). Furthermore, in order to investigate the hypothesis that centre policies or personal feelings might differ if analysed by centre volume (number of live donor nephrectomies (LDNs) performed annually), we divided the European results in the following groups: (0–25 LDNs per year; 26–50, 51–100, 100+) (S4 Table).

### Data collection

The initial invitation to participate with the survey was sent on the 14^th^ of August 2014. Two reminders were sent, each with one month in between.

### Statistical analysis

All analyses were conducted using IBM SPSS Statistics for Windows, Version 21.0 (IBM Corp. Released 2012. Armonk, NY: IBM Corp.). Categorical variables were compared using the Chi-square test and continuous variables were compared with the Mann-Whitney *U* test or the Kruskal-Wallis test. A *P*-value less than 0.05 was considered statistically significant.

## Results

**Fig 1:**
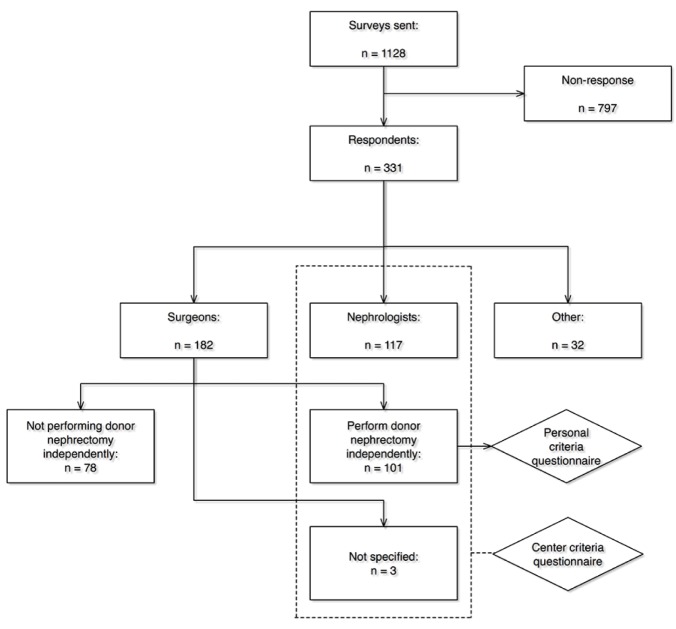
Flow-chart of the inclusion and exclusion of the questionnaire respondents.

The online survey was sent to 1128 ESOT-members of whom 331 (29.3%) completed the survey. 182 respondents were surgeons, 117 were (transplant) nephrologists, and 32 had another professional function. Of the 182 surgeons, 101 performed live donor nephrectomies independently. Three surgeons did not mention whether they performed the operation independently and were excluded from the personal criteria analysis. The remaining 78 surgeons that did not perform nephrectomies independently were excluded from the survey, as well as the 32 transplant professionals who were not a surgeon or nephrologist. After excluding the aforementioned participants, 221 questionnaires were included for analyses. A flow-chart of the process is depicted in Fig 1. It has to be noticed that it could be that several respondents work in the same centre, and thus, may introduce some bias.

### Centre criteria

An overview of all the questions and answers is shown in Table 1. The 187 respondents from Europe where divided as follows: 113 from north-western countries, 55 from Mediterranean countries and 19 from eastern countries. The mean number of live donor kidney transplantations performed amongst the centres of ESOT-members was 40.9 ± 37.5 / year. For Europe, this number was more or less the same. Between regions in Europe, there was a significant difference (*P*<0.001), the highest number of live donor RT was in the north-western transplant centres (55.0 ± 40.5). The mean number of transplantations from deceased donors in ESOT-member centres was 68,9 per year. Respondents in Eastern Europe perform the most transplantations from deceased donors, and Mediterranean countries the least, with a significant difference between regions (*P =* 0.03). Furthermore, significant differences were seen when answers of European respondents were divided based on centre volumes (S4 Table).

**Table 1:** Overview of centre criteria questions.

Mean (SD)						
	World (n = 221)	Europe (n = 187)	Northwest (n = 113)	Mediterranean (n = 55)	East (n = 19)	*P*-value Overall
Kidney TX from a live donor (numbers/region/year)	40.9 (37.5)	40.9 (38.3)	55 (40.5)	18.4 (21.3)	22.6 (21)	**< 0.001**
Kidney Tx from a deceased donor (numbers/region/year)	68.9 (48.8)	80 (47.5)	80.2 (47.8)	62.5 (38.0)	80.6 (65.9)	**0.031**
**Does your centre accept live donors with the following BMI-categories?**						
Overweight	99.5%	99.4%	100%	100%	93.8%	**0.009**
Obesity	69.5%	69.5%	71.3%	64.0%	75.0%	0.579
Morbid Obesity	16.2%	15%	17.8%	12.0%	6.3%	0.378
Morbid Obesity (Class II)	5.1%	4.2%	5.0%	4.0%	0%	0.654
**Does your centre accept minors (<18 years) as live donors?**						
	3.6%	3.6%	5.0%	2.0%	0%	0.472
**Does your centre accept women of childbearing age as live donors?**						
	82.2%	82%	85.1%	80.0%	68.8%	0.257
**Does your centre accept live donors with impaired fasting glucose?**						
	42.9%	41.5%	40.6%	44.9%	35.7%	0.796
**Does your centre accept live donors with hypertension?**						
No	12.2%	10.1%	10.4%	4.1%	28.6%	**0.039**
If controlled 1 agent	47.6%	47.2%	39.6%	65.3%	35.7%	
If controlled 2 agents	32.8%	35.2%	40.6%	24.5%	35.7%	
If controlled with > 2 agents	1.6%	1.3%	2.1%	0%	0%	
Yes	5.8%	6.3%	7.3%	6.1%	0%	
**Does your centre have an upper age limit for live kidney donors?**						
Yes	1.1%	0.6%	1.0%	0%	0%	**0.003**
Yes, max 60	5.3%	1.9%	1.0%	2.0%	7.1%	
Yes, max 65	10.1%	8.2%	4.2%	18.4%	0%	
Yes, max 70	10.6%	10.1%	3.1%	18.4%	28.6%	
Yes, max 75	10.1%	6.9%	7.3%	6.1%	7.1%	
Yes, max 80	5.3%	5%	5.2%	6.1%	0%	
No age limit	57.1%	67.3%	78.1%	49.0%	57.1%	
**Does your centre accept live donors with more than 1 renal artery?**						
No	6.9%	8.2%	2.1%	14.3%	28.6%	**< 0.001**
Yes, max 2 arteries	40.7%	40.3%	31.3%	55.1%	50.0%	
Yes, max 3 arteries	21.7%	20.8%	28.1%	12.2%	0%	
Yes, max 4 arteries	3.2%	2.5%	3.1%	2.0%	0%	
Yes, no maximum	27.5%	28.3%	35.4%	16.3%	21.4%	
**Does your centre accept live donors with more than 1 renal vein?**						
No	9%	10.7%	5.2%	16.3%	28.6%	**0.010**
Yes, no maximum	38.1%	35.8%	29.2%	46.9%	42.9%	
Yes, max 2 veins	20.1%	18.2%	25.0%	8.2%	7.1%	
Yes, max 3 veins	0.5%	0.6%	1.0%	0%	0%	
Yes, max 4 veins	32.3%	34.6%	39.6%	28.6%	21.4%	
**Does your centre accept live donors with kidney stones?**						
No	26.9%	26.9%	21.3%	31.3%	50.0%	0.163
Yes, but only if the remaining kidney is free	54.3%	53.8%	58.5%	52.1%	28.6%	
Yes	18.8%	19.2%	20.2%	16.7%	21.4%	
**Does your centre accept live donors with one or more kidney stones in the contralateral kidney?**						
	14%	12.2%	11.7%	12.5%	14.3%	0.959
**Does our centre accept live donor kidneys with a renal malignancy smaller than 3 cm?**						
	21%	22.4%	30.9%	12.5%	0%	**0.005**
**Does your centre accept live donors with renal cysts?**						
Yes, max Bosniak I	33.9%	32.1%	42.5%	52.7%	36.8%	0.345
Yes, max Bosniak II	39.8%	41.2%	46.9%	40.0%	36.8%	0.566
Yes, max Bosniak IIF	10%	9.6%	9.7%	9.1%	10.5%	0.982
Yes, max Bosniak III	0.5%	0.5%	0%	1.8%	0%	0.299
Yes, max Bosniak IV	0%	0%	0%	0%	0%	-
**Which specialist(s) does a live donor meet during regular screening in your centre?**						
(Transplant) surgeon	70.6%	70.1%	69.9%	78.2%	47.4%	**0.041**
(Transplant) nephrologist	82.8%	81.8%	82.3%	83.6%	73.7%	0.611
Anesthesiologist	46.2%	48.1%	46.0%	58.2%	31.6%	0.105
Social worker	26.7%	22.5%	30.1%	12.7%	5.3%	**0.007**
Nurse practitioner	41.6%	42.2%	52.2%	32.7%	10.5%	**0.001**
Psychologist/Psychiatrist	42.5%	41.7%	36.3%	58.2%	26.3%	**0.009**
Other	15.8%	15.5%	18.6%	12.7%	5.3%	0.264
**Is every live donor discussed in a multidisciplinary team?**						
	90.3%	90.4%	89.4%	89.6%	100%	0.441
**Which specialist(s) are part of the multidisciplinary team of your centre?**						
(Transplant) surgeon	73.8%	72.7%	71.7%	74.5%	73.7%	0.922
(Transplant) nephrologist	74.2%	73.3%	71.7%	76.4%	73.7%	0.812
Anesthesiologist	38.5%	40.1%	32.7%	50.9%	52.6%	**0.039**
Social worker	20.4%	13.4%	15.9%	10.9%	5.3%	0.367
Nurse practitioner	51.6%	48.1%	56.6%	38.2%	26.3%	**0.011**
Psychologist/Psychiatrist	38%	36.9%	26.5%	56.4%	42.1%	**0.001**
Other	21.3%	19.3%	21.2%	16.4%	15.8%	0.695
**Does your centre perform standard pre-operative imaging during the screening of donors?**						
	100%	100%	100%	100%	100%	-
**What modalities of pre-operative imaging are used in your centre?**						
MRI/MRA	15.4%	17.6%	23.0%	9.1%	10.5%	0.059
CT/CTA	73.3%	70.6%	67.3%	78.2%	68.4%	0.337
Invasive angiography	4.1%	2.7%	2.7%	1.8%	5.3%	0.725
Ultrasound	46.6%	45.5%	38.9%	50.9%	68.4%	**0.036**
Other	5.9%	5.3%	8.0%	1.8%	0%	0.138
**Do you perform standard radioisotope renography as part of the live donor screening process?**						
	65.4%	67.7%	60.2%	79.2%	78.6%	**0.049**
**What kind of functional screening do the donors in your centre undergo?**						
MAG-3 scan	35.3%	37.4%	44.2%	23.6%	36.8%	**0.035**
DTPA-scan	28.1%	24.6%	14.2%	45.5%	26.3%	**<0.001**
DMSA-scan	19%	18.2%	19.5%	18.2%	10.5%	0.646
Other	13.6%	13.9%	19.5%	5.5%	5.3%	**0.025**
**What kind of surgical techniques for live donor nephrectomy are practiced in your centre?**						
Open (lumbotomy)	17.6%	15.5%	8.8%	23.6%	31.6%	**0.006**
Open (mini-incision)	25.8%	26.7%	27.4%	23.6%	31.6%	0.769
Laparoscopic transperitoneal	32.1%	29.9%	31.0%	32.7%	15.8%	0.354
HALS	31.2%	29.9%	31.9%	27.3%	26.3%	0.777
Retroperitoneoscopic–no hand-assistance	5.4%	5.3%	6.2%	5.5%	0%	0.539
HARP	15.8%	17.1%	25.7%	3.6%	5.3%	**0.001**
Robot-assisted laparoscopic transperitoneal	8.6%	10.2%	10.6%	12.7%	0%	0.276
Other	2.3%	1.1%	1.8%	0%	0%	0.516

SD: standard deviation. Tx: transplantation, BMI: Body Mass Index, MAG-3 scan: Mercaptoacetyltriglycine-scan, DTPA-scan: Diethylene Triamine Pentacaetic Acid-scan, DMSA-scan: dimercaptosuccinic acid-scan, HALS: Hand-assisted laparoscopic transperitoneal, HARP: Hand-assisted retroperitoneoscopic laparoscopic.

#### Overweight and obesity

99.5% of the respondents’ centres accept donors with overweight (>25, <30), 69.5% accept obese donors (BMI 30–35), morbidly obese donors are considered in 16.2% of the centres, and only 5.1% accept donors with a BMI higher than 40. No significant differences were seen between regions in accepting obese live kidney donors.

#### Hypertension

Donors with hypertension are accepted in 5.8% of the respondents’ centres, even if not controlled with antihypertensive medication; 47.6%, 32.8% and 1.6% of the centres accept these donors if the hypertension is controlled with 1, 2, or more than 2 agents, respectively. 12.2% of the centres decline donors with hypertension, regardless if well controlled with medication. A significant difference exists between regions in Europe (*P* = 0.04).

#### Older donors

57.1% of the respondents’ centres have no upper age limit for live kidney donors. 1.1% of the centres have an undefined age limit. Again, a significant difference is seen between centres in Europe (*P*<0.01).

#### Vascular multiplicity

Arterial multiplicity and venous multiplicity are not considered a contra-indication in 93.1% and 91.0% of the respondents’ centres, respectively. In Europe, it seems that Mediterranean and Eastern centres have more strict policies regarding vascular multiplicity, with significant differences both in arterial as in venous multiplicity.

#### Renal malignancies

Renal malignancies smaller than 3 cm are accepted by 21% of the respondents’ centres. North-western centres have the highest acceptance rate of these donors, versus 0% of the eastern centres (*P*<0.01).

#### Multidisciplinary teams

In 90.3% of the respondents’ transplant centres, every donor is discussed in a multidisciplinary team. We wondered if the composition of the specialists that a potential living kidney donor meets in context of a regular screening differs between regions. Interestingly, there are significant differences in specialists that screen donors between European regions. Thirty-five respondents filled out that their donors are also screened or seen by other professionals than the ‘standard’ list as part of regular screening, amongst which ward nurses, transplant coordinators, urologists, cardiologists, independent donor advocates, health educators, members of an ethical committee, and sometimes even an endocrinologist or gynaecologist. Forty-seven participants responded that also other professionals are part of the multidisciplinary team, amongst which transplant coordinators, radiologists, immunologists, geneticists, cardiologists and urologists. Regarding the composition of this team, significant differences are also seen between regions in Europe. Of the centres that do not discuss every donor in a multidisciplinary team (9.6%), only the (transplant) surgeon or nephrologist decides on the final acceptance of the donor.

#### Imaging and functional scanning

All respondents’ transplant centres perform standard imaging during the screening process. Most of the centres (73.3%) use CT or CTA as imaging modality to assess the renal anatomy. Following CT, ultrasound is used in 46.6% of the centres, however not as the only modality (mostly in combination with CT). Only in the use of ultrasound, a significant difference was found between European centres, where North-western centres use ultrasound least frequently, and centres in Eastern countries the most. The respondents that filled out that other types of imaging are used misinterpreted functional screening methods like radioisotope renography with imaging techniques.

Standard radioisotope renography is performed in 65.4% of the respondents’ centres, of which a MAG3-scan is performed in 35.3% of these centres. In Mediterranean centres, radioisotope renography is used the most, compared to other European regions (*P* = 0.05). There are some differences in the use of several modalities available between regions. Reasons for not performing standard radioisotope renography are that imaging modalities seem sufficient, only if there is a significant size discrepancy between the two kidneys, or other reasons to suspect the relative functional contribution of each kidney to total renal function is different. Other functional screening modalities mentioned were EDTA- or iohexol clearance calculations.

#### Surgical techniques of live donor nephrectomy

Although laparoscopic donor nephrectomy[8] is considered as golden standard in most of the transplant centres, still several other (or newly developed) techniques are practiced. Despite the outcome of some high-quality randomized controlled trials and meta-analyses,[9–11] the open technique via a lumbotomy or mini-incision[12] is used in 17.6% and 25.8% of the centres, respectively. The open technique is mostly performed in Eastern centres (31.6%) and the least in north-western centres (8.8%). 31.2% perform the hand-assisted laparoscopic technique,[13] and 15.8% the hand-assisted retroperitoneoscopic technique.[14] Pure retroperitoneoscopic approaches,[15] and robot-assisted laparoscopic donor nephrectomy[16] are practiced in 5.4% and 8.6% of the respondents’ centres, respectively.

#### Other

For other results and differences that were not different between regions, we refer to Table 1.

### Personal criteria

In total, 101 of the 182 surgeons that performed donor nephrectomies independently, filled out the personal criteria questionnaire, consisting of 85 surgeons working in European centres amongst which were 64 surgeons from North-western centres, 17 from Mediterranean and 4 from Eastern centres. Medians and ranges of the five-point Likert scale are presented in Table 2. Furthermore, regarding several outcome measures, significant differences were seen between continents and also when answers were divided based on centre volumes (S4 Table).

**Table 2:** Overview of personal criteria questions.

Median + ranges	World (n = 101)	Europe (n = 85)	Northwest (n = 64)	Mediterranean (n = 17)	East (n = 4)	p-value
**How likely is it that you would personally perform a live donor nephrectomy in a donor who is:**						
Overweight (25–30)	5 (2–5)	5 (2–5)	5 (3–5)	4 (2–5)	5 (4–5)	**0.002**
Obese (30–35)	3 (1–5)	3 (1–5)	4 (1–5)	3 (1–5)	4 (3–5)	0.062
Morbidly obese (35–40)	2 (1–5)	2 (1–5)	2 (1–5)	1 (1–4)	2 (1–2)	0.158
Morbidly obese (40 +)	1 (1–5)	1 (1–5)	1 (1–5)	1 (1–3)	1 (1–1)	0.166
Upper age limit for LKD	60 (60-no age limit)	No age limit (no age limit-other)	No age limit(no age limit-other)	70 (no age limit-other)	70 (no age limit-other)	0.112
Minors as donors	1 (1–5)	1 (1–5)	1 (1–5)	1 (1–4)	1 (1–1)	0.153
Women of childbearing age	4 (1–5)	4 (1–5)	4 (1–5)	3 (2–5)	4 (2–4)	0.254
Impaired fasting glucose	2 (1–5)	2 (1–5)	2 (1–5)	2 (2–4)	2 (1–2)	0336
**How likely is it that you would personally accept a donor with hypertension with the following conditions?**						
Without agents	3 (1–5)	3 (1–5)	3 (1–5)	3 (1–5)	4 (2–5)	0.471
If well controlled with 1 agent	4 (1–5)	4 (1–5)	4 (1–5)	4 (2–5)	4 (1–5)	0.257
If well controlled with 2agents	2 (1–5)	2 (1–5)	2 (1–5)	2 (1–4)	3 (1–4)	0.739
If well controlled with >2agents	1 (1–5)	1 (1–5)	1 (1–5)	1 (1–2)	1 (1–2)	0.816
**What is in your opinion more important, the arterial or the venous anatomy?**						
	Artery	Artery	Artery	Artery	Equally important	0.636
**How likely is it that you would personally accept a donor with the following number of renal arteries?**						
1 renal artery	5 (3–5)	5 (3–5)	5 (5–5)	5 (4–5)	5 (5–5)	0.135
2 renal arteries	5 (2–5)	5 (2–5)	5 (2–5)	4 (2–5)	5 (4–5)	**0.001**
3 renal arteries	3 (1–5)	3 (1–5)	3 (1–5)	2 (1–4)	2 (2–4)	0.109
4 renal arteries	2 (1–5)	2 (1–5)	2 (1–5)	1 (1–4)	1 (1–2)	0.236
>4 renal arteries	1 (1–5)	1 (1–5)	1 (1–5)	1 (1–3)	1 (1–2)	0.735
**How likely is it that you would personally accept a donor with the following number of renal veins?**						
1 renal vein	5 (3–5)	5 (3–5)	5 (4–5)	5 (4–5)	5 (5–5)	0.557
2 renal veins	5 (1–5)	5 (1–5)	5 (2–5)	5 (1–5)	5 (4–5)	**0.018**
3 renal veins	4 (1–5)	4 (1–5)	4 (1–5)	4 (1–5)	2 (1–3)	**0.049**
4 renal veins	2 (1–5)	2 (1–5)	2 (1–5)	2 (1–4)	1 (1–3)	0.299
>4 renal veins	2 (1–5)	2 (1–5)	2 (1–5)	2 (1–4)	1 (1–3)	0.867
**How likely is it that you would personally accept a kidney with stones for donation?**						
Kidney with stones for donation	3 (1–5)	3 (1–5)	4 (1–5)	3 (1–4)	2 (1–5)	0.179
Stone(s) in contralateral kidney	2 (1–5)	2 (1–5)	2 (1–5)	2 (1–3)	2 (1–2)	0.281
**Which technique(s) do you** **preferably** **use for live donor nephrectomy?**						
Open lumbotomy	6.9%	5.9%	3.1%	5.9%	50.0%	**0.001**
Open (mini-incision)	19.8%	20%	17.2%	29.4%	0%	0.319
Laparoscopic transperitoneal	36.6%	32.9%	34.4%	35.3%	0%	0.356
HALS	31.7%	31.8%	31.3%	35.3%	25.0%	0.909
Retroperitoneoscopic, no hand-assistance	5.9%	4.7%	6.3%	0%	0%	0.502
HARP	19.8%	21.2%	28.1%	0%	0%	**0.024**
Robot-assisted laparoscopic transperitoneal	4.0%	4.7%	1.6%	17.6%	0%	**0.019**
Other	3.0%	2.4%	3.1%	0%	0%	0.715
**What is your percentage of refusal for potential live kidney donors?**						
	20–30% (0%– 60%)	10–20% (0%– 60%)	20–30% (0%—other)	20–30% (0%– 60%)	20–30% (10%—other)	0.747
**Do you sometimes deviate from your centre policy?**						
	23.7%	23.4%	27.6%	13.3%	0%	0.315
**Regarding which patient characteristic do you deviate?**						
Weight	77.3%	72.2%	18.8%	5.9%	-	0.199
Blood pressure	36.4%	27.8%	7.8%	0%	-	0.234
Older age	31.8%	16.7%	3.1%	5.9%	-	0.593
Younger age	4.5%	5.6%	1.6%	0%	-	0.604
Women of childbearing age	13.6%	11.1%	3.1%	0%	-	0.460
Impaired fasting glucose	36.4%	33.3%	7.8%	5.9%	-	0.787
Vascular multiplicity	27.3%	27.8%	7.8%	0%	-	0.234

SD: standard deviation, Tx: transplantation, BMI: Body Mass Index, MAG-3 scan: Mercaptoacetyltriglycine-scan, DTPA-scan: Diethylene Triamine Pentacaetic Acid-scan, DMSA-scan: dimercaptosuccinic acid-scan, HALS: Hand-assisted laparoscopic transperitoneal, HARP: Hand-assisted retroperitoneoscopic laparoscopic.

#### Vascular multiplicity

In all regions, surgeons replied that they consider the arterial renal anatomy to be more important than the venous anatomy, except for the Eastern centres, where the renal and the venous anatomy are considered equally important. Regarding vascular multiplicity, as expected, surgeons are less likely to accept a donor with more arteries or veins, not significantly different between regions for the arterial anatomy. As for the venous anatomy however, there is some difference between regions in selection of donors with two or three renal veins (*P* = 0.02 and *P*<0.05)

#### Preferred surgical techniques of donor nephrectomies

As presented in the centre-criteria part of the survey, still 17% of the centres perform the lumbotomy for graft retrieval, and about a quarter the mini-open technique. These techniques seem to be less preferred, showing that the laparoscopic transperitoneal is the most favourite technique, closely followed by the hand-assisted technique. The relatively newer techniques are significantly less preferred in Eastern centres, in contrast to the open lumbotomy technique, which is preferred in 50% of the Eastern centres (*P<*0.001). This could be attributed by the fact that these centres might not have access to these techniques. 26.3% of the respondents chose more than one technique as preferable.

#### Decline of potential living kidney donors

In total, between 20 and 30% of the participants sometimes decline a potential live kidney donor. There is no significant difference between continents. The top three reasons for declining were: hypertension (12.1%), glucose levels (diabetes) (10.5%) and renal (dys)function (9.7%). Other reasons for refusal of a potential donor were: anatomy, overweight/obesity or other comorbidities. Participants were also asked if they maintain other criteria, which were not previously mentioned to refuse a donor. Several criteria were mentioned; psychological reasons, ethical uncertainty, or uncertainty of the motivation of the potential donor. No differences were seen between centres in Europe.

#### Deviation from centre policy

Interestingly, 23.7% of the surgeons (sometimes) deviate from their centre policy regarding acceptance of extended live kidney donor criteria. Between regions in Europe, there seems to be no significant difference, although none of the surgeons in Eastern countries deviate from their centre policy. Body weight was the criterion that was most frequently mentioned as reason to deviate from centre policy (77.3%). No differences exist between centres in Europe. Interestingly, however, a significant difference was seen between continents regarding ‘older age’ as reason to deviate from the centre policy (*P*<0.01, S3 Table). American transplant surgeons are much more likely to deviate from their centre policy regarding age than European centres.

#### Other

For other results and differences that were not different between regions, we refer to Table 2.

## Discussion

Whether it is truly safe for extended criteria donors to donate a kidney during live will be determined largely by long-term follow up data. In general, these long-term data of living kidney donors are not yet available. Although several studies have been published regarding ‘medium’-term outcome, the longest follow-up data available is with a follow up time of around thirty years.[17] Most studies report excellent outcome after live donor nephrectomy in short-term follow-up,[5,17–19] however recent studies are more reluctant towards the trend to accept any healthy individual as a live kidney donor, since their risk on end-stage renal disease might not be comparable to the general population, and could be even higher.[20–22] As the WHO states that, ‘Live donations are acceptable when the donor’s informed and voluntary consent is obtained, when professional care of donors is ensured and follow-up is well organized, and when selection criteria for donors are scrupulously applied and monitored’,[23] it is important that transplant professionals give accurate information regarding possible complications. Unfortunately, there is no worldwide consensus regarding the informed consent procedure.[24] Especially in the case of extended criteria donors, long-term follow-up data is lacking, as these donors have been increasingly accepted in the last decade. We know that donors with overweight/obesity,[25] and donors with vascular multiplicity have good short-term outcome.[26–28] However, regarding other extended criteria, there is still a lot of uncertainty.

Based on the results of the questionnaire, acceptance of donors with overweight or obesity is fairly comparable. From earlier studies we know that outcome of both lean as overweight/obese donors are comparable.[3,29] Some centres accept minors as potential donors, which is an interesting phenomenon, as current guidelines state that minors should only be considered as potential donors if no other options exist, mostly in case of identical twins. There seems to be no reluctance in accepting a woman of childbearing age for kidney donation, which is in line with the Amsterdam Forum criteria, stating that donor nephrectomy is not detrimental to the prenatal course or outcome of future pregnancies.[30] However, recent literature recommends a more careful approach regarding this group of potential donors.[22], given the slightly increased risk on preeclampsia.

Regarding impaired fasting glucose in a potential donor, about half of the centres have no objection, which is in contrast with guidelines.[30] Remarkably, 5.8% of the centres consider a donor with hypertension that is uncontrolled, and 6.3% of European centres even accepts these donors. This is an interesting finding, as guidelines are more or less unanimous; uncontrolled hypertension should be considered as a contra-indication for donation.[30,31]

We know from previous studies that older living donors have excellent outcome,[32,33] luckily, more than half of the centres have no age limit for the donors. However, there are differences between regions in Europe. In America, there seems to be a more strict policy regarding donor age (S3 Table). One of the main questions is whether a kidney from a 70-year old will last as long as that from a 50-year old donor. Furthermore, many guidelines have not included statements regarding older age.[30,34,35] The British guidelines however state that older age is not an absolute contra-indication for donation, but that the medical work-up of older donors must be particularly rigorous to ensure suitability. They also mention that the older donor may have a greater risk of developing perioperative complications.[31] Ahmadi *et al*.[3] performed a systematic review, combining all available evidence to date, stating that older age does not seem to have a negative impact on the outcome after donor nephrectomy.

More than a quarter of the centres have no maximum for vascular multiplicity, however, geographical differences exist. The eastern countries in Europe as well as American centres seem to be more reluctant. As we know that about a quarter of the general population has vascular multiplicity,[36,37] we lose a considerable number of potential donors if these are excluded. Previous studies have shown that both donors as recipients from kidneys with vascular multiplicity have excellent outcome, at least with arterial multiplicity up to three renal arteries.[3,38] The questions about cysts, kidney stones and renal malignancies in donor kidneys give no striking results, although Eastern transplant centres do not accept kidneys with renal malignancies. Regarding kidney stones, the guidelines state that an asymptomatic potential donor with a current (or a history of) single stone can be safely selected for donation, but such potential kidney donors should be screened for metabolic stone forming abnormalities.[30,31] However, 27% of the centres decline a donor with stones, even if the contralateral kidney is free of stones.

In Europe, full transperitoneal laparoscopic donor nephrectomy is the most popular technique among other different (and new) techniques in centres [39,40]. The currently available techniques for minimally invasive live donor nephrectomy are safe and associated with low complication rates, and minimal risk of mortality. [41]

Almost no differences are seen between the personal criteria and the centre criteria part of the questionnaire. Interestingly, European surgeons seem to be more reluctant in accepting women of childbearing age than American surgeons (S3 Table). Little literature is available regarding outcome in this group, with varying results.[42,43] A recent publication by Garg *et al*.[22] however, reports a higher incidence of gestational hypertension and preeclampsia in this group of donors. However, the absolute risks are small, and the severity of the preeclampsia very mild. Another important issue is the percentage of donor decline. Overall, around 20–30% of the donors are excluded, for reasons previously mentioned, although we are not sure exactly when in the screening process these donors are declined.

One of the most daring questions was whether a respondent sometimes deviates from centre policy. A quarter of the surgeons sometimes deviate, mostly if the possible donor is overweight or obese. This could be explained by the fact that the BMI does not take the fat distribution into account, and that an obese donor might be perfectly suitable in terms of surgical difficulty. American surgeons seem to be more inclined to overrule their centre policy regarding the age limit of a possible donor. This significant difference can be attributed to the fact that American centres hold stricter age limit policies. It is a somewhat striking result that 25% of the professionals deviate, and perhaps we should be more careful including extended criteria live kidney donors, as we do not have long-term follow-up data of these specific donors.

Regarding centre volumes (S4 Table), not unexpectantly, we found that with higher volumes, professionals are less reluctant to accept extended criteria donors. We observed this phenomenon across all types of extended criteria (obesity, age, hypertension and numbers of vessels).

### Limitations

The response percentage of this survey was 29.3%, which could be considered low. However, in Europe, there is no (public) database available of transplant professionals. Luckily, the ESOT- database could generate a list of all members, and sort them by field of interest and specific profession. At that time, we felt that this would be the only viable option to send out a survey and get the best possible response in terms of absolute numbers, deliberately accepting a possible large number of non-respondents because of not updated contact information by members, or not having selected the right profession or interest. As mentioned in the methods section, the anonymity prohibited us from analysing or pooling centre data. Since we felt that professionals otherwise could be hesitant to fill out the questionnaire, we accepted this limitation. Therefore, one can argue that found p-values might not be of less value. Nonetheless, to enhance the insight of the data, we decided to state these values, being aware of the necessity of careful interpretation. Furthermore, not all transplant centres around the world have a live kidney donation program, and are therefore less likely to respond. In our opinion, the absolute number of response (n = 331) is a high number, being a good representative of transplant professionals around Europe. We received some responses from America (n = 13) and Asia (n = 5), because not only European professionals are members of ESOT. It is possible though, that the non-European ESOT members represent a special subgroup (might be Europeans that emigrated to America and Asia, for example). Although the focus of our survey is on Europe, we decided to make these data available in the supplemental data. We should also consider the different cultural behaviours and socio-economic status of the participating countries. It could be that these differences are attributing to the results, and should therefore be carefully interpreted. An idea for a follow-up questionnaire would be to ask about the awareness of the respondents of the KDIGO Guidelines on live kidney donation (still in draft), and if those were adhered to.[44]

Concluding, in this era of organ shortage, extended criteria donors are increasingly considered as candidates for live kidney donation. There is still great discrepancy between available guidelines, literature and as we now know, attitudes of transplant professionals regarding extended criteria donors. Guidelines are very superficial regarding some extended criteria, over the last years however, more and more literature has become available, showing good short-term outcome of extended criteria donors. Perhaps the aim should not be to have the same acceptance criteria worldwide, as some centres might be reluctant because of relatively low volume. However, considering the inequity to live donor kidney transplantation for kidney patients across Europe, patients and professionals should at least be aware of the possibilities. Although we should bear in mind that long-term outcome of these donors still should be unravelled, it is clear that (based on the results of this survey), transplant professionals are prepared to accept these donors. We also strongly advocate for a life-long follow up of live kidney donors to minimize the long-term risks and enable early interventions.[7,45] By performing this survey, we aimed to give the transplant community more insight in their policies and attitudes, hopefully leading to an eventual consensus regarding extended criteria donors, and thereby enlarging the donor pool.

## Supporting information

S1 Table Questionnaire. (DOCX)

S2 Table Division into European centers. (DOCX)

S3 Table Worldwide data. (DOCX)

S4 Table Analyzed by center volume (number of annual live kidney donations). (DOCX)

## Abbreviations

BMI: Body Mass Index
ESOT: European Society for Organ Transplantation
ESRD: End-stage renal disease
MAG3-scan: mercaptoacetyltriglycine-scan
LDNs: Live donor nephrectomies
LESS: Laparo-Endoscopic Single-Site Surgery
NOTES: Natural orifice transluminal endoscopic surgery
WHO: World Health Organization
