# Supplementary material for: Attitudes among transplant professionals regarding shifting paradigms in eligibility criteria for live kidney donation
Source: PLoS One. 2017 Jul 21;12(7):e0181846. doi: 10.1371/journal.pone.0181846 (PMC5521829; doi:10.1371/journal.pone.0181846)
Supplement: S1 Table — (DOCX) [file pone.0181846.s001.docx]

**S1. Table: Questionnaire**

**Introduction Survey**In the questionnaire below, you will find several questions regarding standard versus extended criteria for live kidney donors. The survey consists of two separate parts.
1) The policy of your **center** towards acceptance of these donor types.
2) Your **personal opinion** on acceptance of these donor types.

Please indicate your professional title.
**Surgeon**
*If you ticked ‘Surgeon’, please indicate whether you perform live donor nephrectomies independently.*
*Yes, I perform live donor nephrectomies independently
No, I do not perform live donor nephrectomies independently.

If you answered ‘No, I do not perform nephrectomies on my own’ please* ***only*** *fill out part 1, Center Criteria.*

**Transplant nephrologist**

***None of the above*** *If you ticked ‘None of the above’, the survey ends here for you. Many thanks.*

If you have no objection please write down your email address below to contact you should further questions arise.
*(If you would like to remain anonymous you can leave this field blank)*Email address: ………………………………………………………………………………………………………………………………………..

**CENTER Donor selection criteria**

| 1. What is your age? | *……………………………………………………………..* |
| --- | --- |
| 2a. How many live donor kidney transplantations are carried out in your center per year?  2b. How many kidney transplantations from **deceased** donors are carried out in your center per year? | ……………………………………………………………..  …………………………………………………………….. |
| 3. Does your center accept live kidney donors with the following Body Mass Indices?  Overweight (BMI ≥25 and <30) Obesity (BMI ≥30 and <35) Morbid obesity (BMI ≥35 and <40)  BMI ≥ 40 | Yes No  Yes No Yes No Yes No |
| 4. Does your center accept minors as live kidney donors  (Age <18 years) | Yes No |
| 5. Does your center accept women of childbearing age as live kidney donors? | Yes No |
| 6. Does your center accept live kidney donors with impaired fasting glucose?  *(Defined as: fasting plasma glucose of 100 to 125 mg/dL)* | Yes  No |
| 7. Does your center accept donors with hypertension (defined as systolic blood pressure ≥140mmHg and diastolic ≥90mmHg) | Yes  Yes, if well controlled with 1 agent  Yes, if well controlled with 2 agents Yes, if well controlled with ≥ 2 agents  No |
| 8. Does your center use an upper age limit for live kidney donors? | Yes  Yes, maximum of 60  Yes, maximum of 65  Yes, maximum of 70  Yes, maximum of 75  Yes, maximum of 80  Yes, other : ………….. years  No age limit |
| 9a.Does your center accept live kidney donors with more than 1 renal **artery?**  9b**.** Does your center accept live kidney donors with more than 1 renal **vein?** | No, maximum of 1 renal artery  Yes, maximum of 2 renal arteries  Yes, maximum of 3 renal arteries  Yes, maximum of 4 renal arteries  No, maximum of 1 renal vein  Yes, maximum of 2 renal veins  Yes, maximum of 3 renal veins  Yes, maximum of 4 renal veins |
| 10. Does your center accept a kidney with stones for live kidney transplantation? | Yes No  Yes, but only if the remaining kidney is free of stones |
| 12. Does your centre accept donors with a renal malignancy < 3 cm if the tumor can be resected during bench surgery? | Yes No |
| 13. What Bosniak classification does your center accept in a donor with a renal cyst? | Bosniak I: Malignancy risk less than 1%  Bosniak II: Malignancy risk less than 3%  Bosniak IIF: Malignancy risk 5-10%  Bosniak III: Malignancy risk 40-60%  Bosniak IV: Malignancy risk >80% |
| 14. Which specialists does a potential living kidney donor meet in the context of a regular screening? | (Transplant) surgeon  (Transplant) nephrologist  Anesthesiologist  Social worker  Nurse practitioner  Psychologist/ Psychiatrist  Other, namely:  *……………………………………………………………..* |
| 15a. Is every potential donor discussed in a multidisciplinary team?  *If your donor is* ***not*** *discussed in a multidisciplinary team please answer the following question.*  15b. How or by whom is decided whether the donor is eligible for live kidney donation? | Yes No  *If the answer is ‘Yes’, please tick off which specialists are included in the multidisciplinary team.  Only if your answer is ‘No’, please fill out 15b.* (Transplant) surgeon(s)  *(*Transplant) nephrologist(s)  Anesthesiologist (s)  Social worker(s)  Nurse practitioner(s)  Psychologist(s)/ Psychiatrist(s)  Other, namely:  *……………………………………………………………..*  *………………………………………………………....* |
| 16. Do you perform standard imaging of the kidneys as part of the live donor screening process? | Yes No  *If the answer is ‘Yes’, please tick off which type of radiological imaging is used in your centre.* MRI/MRA CT/CT-A  Invasive angiography  Ultrasound  Other, namely*: ……………………………………………………………...*  If no, why not?  *……………………………………………………………...* |
| 17. Do you perform standard radioisotope renography as part of the live donor screening process? | Yes No  *If the answer is ‘Yes’, please tick off what functional screening is used in your centre.*  MAG3-scan  DTPA-scan  DMSA-scan  Other, namely: ………………………………………………………………  If no, why not?  *……………………………………………………………...* |
| 18.Which techniques for live donor nephrectomy **are practiced**  in your center? | Open (Lumbotomy)  Open (Mini-incision)  Laparoscopic transperitoneal  Hand Assisted Laparoscopic transperitoneal  Retroperitoneoscopic, no hand-assistance  Hand Assisted retroperitoneoscopic  Robot-assisted laparoscopic transperitoneal  Other, namely:  ……………………………………………………………… |

**PERSONAL donor selection criteria**

| 1. How likely is it that you would personally perform a live donor nephrectomy in a donor who is:   Overweight (BMI ≥25 and <30)  Highly unlikely Unlikely  Neutral Likely Very likely  Obese (BMI ≥30 and <35)  Highly unlikely Unlikely  Neutral Likely Very likely  Morbidly obese (BMI ≥35 and <40)  Highly unlikely Unlikely  Neutral Likely Very likely  Morbidly obese (BMI > 40)  Highly unlikely Unlikely  Neutral Likely Very likely | |
| --- | --- |
| 2. How likely is it that you would personally accept a minor as a live kidney donor (Age <18 years)  Highly unlikely Unlikely  Neutral Likely Very likely | |
| 3.What would be an acceptable upper age limit for live kidney donation?  maximum of 60  maximum of 65  maximum of 70  maximum of 75  maximum of 80  other : ………….. years  No age limit | |
| 4. How likely is it that you would personally accept women of childbearing age as live kidney donors?  Highly unlikely Unlikely  Neutral Likely Very likely | |
| 5. How likely is it that you would personally consider potential donors with impaired fasting glucose (plasma glucose of 100 to 125 mg/dL) or previous diabetes, however momentarily under control with drugs?  Highly unlikely Unlikely  Neutral Likely Very likely | |
| 6. How likely is it that you personally accept a donor with hypertension (defined as systolic blood pressure ≥140mmHg and diastolic ≥90mmHg) with the following conditions?  Hypertension without agents:  Highly unlikely Unlikely  Neutral Likely Very likely  Hypertension, well controlled with 1 agent:  Highly unlikely Unlikely  Neutral Likely Very likely  Hypertension, well controlled with 2 agents: Highly unlikely Unlikely  Neutral Likely Very likely  Hypertensions, well controlled with ≥ 2 agents:  Highly unlikely Unlikely  Neutral Likely Very likely | |
| 8. What is in your opinion more important in live kidney donation, the renal arterial or renal venous anatomy?  Artery Vein Equally important | |
| 9.How likely is it that you would personally accept a donor with the following number of arteries?  **1 renal artery**  Highly unlikely Unlikely  Neutral Likely Very likely  **2 renal arteries**  Highly unlikely Unlikely  Neutral Likely Very likely  **3 renal arteries**  Highly unlikely Unlikely  Neutral Likely Very likely  **4 renal arteries**  Highly unlikely Unlikely  Neutral Likely Very likely  **> 4 renal arteries**  Highly unlikely Unlikely  Neutral Likely Very likely | |
| 10. How likely is it that you would personally accept a donor with the following number of veins?  **1 renal vein**  Highly unlikely Unlikely  Neutral Likely Very likely  **2 renal veins**  Highly unlikely Unlikely  Neutral Likely Very likely  **3 renal veins**  Highly unlikely Unlikely  Neutral Likely Very likely  **4 renal veins**  Highly unlikely Unlikely  Neutral Likely Very likely  **>4 renal veins**  Highly unlikely Unlikely  Neutral Likely Very likely | |
| 11. How likely is it that you would personally accept a kidney with stones for donation?  Highly unlikely Unlikely  Neutral Likely Very likely | |
| 12. How likely is it that you would personally accept a kidney from a donor who has stones in the remaining kidney?  Highly unlikely Unlikely  Neutral Likely Very likely | |
| 13.Which technique(s) do you **preferably** use for live donor nephrectomy? *(More than one answer possible)* | Open (lumbotomy)  Open (Mini-incision)  Laparoscopic transperitoneal  Hand Assisted Laparoscopic transperitoneal  Retroperitoneoscopic, no hand-assistance  Hand Assisted Retroperitoneoscopic  Robot-assisted laparoscopic transperitoneal  Other, namely:  ………………………………………………………. |
| 14. What is your percentage of refusal for potential live kidney donors  14b. Please indicate your top 3 reasons for refusal: | *…………………………………………………………*  *1. ……………………………………………………*  *2. ..…………………………………………………*  *3. …………………………………………………..* |
| 15. Do you personally maintain other criteria (which are not previously mentioned ) to refuse a donor? |  |
| 16. Do you sometimes deviate from your center policy? | Yes  No |
| 17. You answered that you sometimes deviate from your center policy. Regarding which patient characteristic do you deviate? | Weight (Overweight or obese)  Blood pressure (hypertension)  Age (older age)  Age (younger age)  Women of childbearing age (if center policy advices to deny these potential donors)  Donors with impaired fasting glucose (if center policy advices to deny these potential donors)  Vascular multiplicity (more than the number of arteries/veins your centery policy advises to accept) |
