# Supplementary material for: Attitudes among transplant professionals regarding shifting paradigms in eligibility criteria for live kidney donation
Source: PLoS One. 2017 Jul 21;12(7):e0181846. doi: 10.1371/journal.pone.0181846 (PMC5521829; doi:10.1371/journal.pone.0181846)
Supplement: S2 Table — (DOCX) [file pone.0181846.s002.docx]

**S2. Table. Division into European centers**

| **Europe (n = 283)** | | |
| --- | --- | --- |
| **Northwest (n = 175)** | **Mediterranean (n = 77)** | **East (n = 31)** |
| United Kingdom (46) | Italy (35) | Poland (8) |
| The Netherlands (45) | France (16) | Hungary (5) |
| Germany (32) | Spain (14) | Czech Republic (4) |
| Belgium (17) | Greece (6) | Estonia (2) |
| Sweden (9) | Portugal (5) | Croatia (2) |
| Austria (7) | Belarus (1) | Latvia (2) |
| Switzerland (6) |  | Lithuania (2) |
| Finland (4) |  | Romania (2) |
| Norway (4) |  | Albania (1) |
| Denmark (3) |  | Macedonia (1) |
| Scotland (2) |  | Moldavia (1) |
|  |  | Slovenia (1) |
