# Supplementary material for: Attitudes among transplant professionals regarding shifting paradigms in eligibility criteria for live kidney donation
Source: PLoS One. 2017 Jul 21;12(7):e0181846. doi: 10.1371/journal.pone.0181846 (PMC5521829; doi:10.1371/journal.pone.0181846)
Supplement: S3 Table — (DOCX) [file pone.0181846.s003.docx]

**S3. Table. Worldwide**

| Mean (SD) |  |  |  |  |  |  |
| --- | --- | --- | --- | --- | --- | --- |
|  | **Total  (n= 221)** | **Europe (n= 187)** | **America  (n= 13)** | **Asia  (n= 16)** | **Other  (n= 5)** | **P-value Overall** |
| Kidney Tx from live donor (numbers/center/year) | 40,9 (37,5) | 40,9 (38,3) | 41 (25,5) | 44,9 (40,6) | 25 (20,8) | 0.770 |
| Kidney Tx from deceased donor (numbers/center/year) | 68,9 (48,8) | 80 (47,5) | 56 (63) | 25,4 (22,2) | 22,5 (28,7) | 0.000 |
| **BMI** | | | | | |  |
| Overweight | 99,5% | 99,4% | 100% | 100% | 100% | 0.981 |
| Obesity | 69,5% | 69,5% | 50% | 87,5% | 50% | 0.175 |
| Morbid Obesity | 16,2% | 15% | 20% | 18,8% | 50% | 0.293 |
| Morbid Obesity (Class II) | 5,1% | 4,2% | 10% | 6,3% | 25% | 0.249 |
| **Minors as donors** | 3,6% | 3,6% | 0% | 6,3% | 0% | 0.836 |
| **Women of childbearing age** | 82,2% | 82% | 80% | 87,5% | 75% | 0.922 |
| **Impaired fasting glucose** | 42,9% | 41,5% | 50% | 56,3% | 25% | 0.568 |
| **Hypertension** | | | | | |  |
| No  If controlled 1 agent  If controlled 2 agents  If controlled with > 2 agents  Yes | 12,2% 47,6% 32,8% 1,6%  5,8% | 10,1%  47,2%  35,2%  1,3%  6,3% | 40% 40%  10%  0%  10% | 18,8%  50%  31,3%  0%  0% | 0%  75%  0%  25%  0% | 0.006 |
| **Upper age limit** | | | | | |  |
| Yes Yes, max 60 Yes, max 65 Yes, max 70 Yes, max 75 Yes, max 80 No age limit | 1,1% 5,3% 10,1%  10,6%  10,1%  5,3%  57,1% | 0,6%  1,9%  8,2%  10,1% 6,9%  5% 67,3% | 10%  30%  30%  0%  30%  0%  0% | 6,3%  25%  12,5%  25%  18,8%  6,3%  6,3% | 0%  0%  25%  0%  50%  25%  0% | 0.000 |
| **More than 1 renal artery** | | | | | | |
| No  Yes, max 2 arteries  Yes, max 3 arteries  Yes, max 4 arteries  Yes, no maximum | 6,9%  40,7%  21,7%  3,2%  27,5% | 8,2%  40,3%  20,8%  2,5%  28,3% | 0%  40%  30%  20%  10% | 0%  37,5%  31,3%  0%  31,3% | 0% 75%  0%  0%  25% | 0.166 |
| **More than 1 renal vein** | | | | | | |
| No  Yes, max 2 veins  Yes, max 3 veins  Yes, max 4 veins  Yes, no maximum | 9%  38,1%  20,1%  0,5% 32,3% | 10,7%  35,8%  18,2%  0,6%  34,6% | 0%  50%  50%  0%  0% | 0%  43,8 25%  0%  31,3% | 0%  75%  0%  0%  25% | 0.251 |
| **Kidney stones** | | | | | | |
| No  Yes, but only if the remaining kidney is free  Yes | 26,9%  54,3%  18,8% | 26,9%  53,8%  19,2% | 40%  50%  10% | 12,5%  62,5%  25% | 50%  50%  0% | 0.620 |
| Stone(s) in contralateral kidney | 14% | 12,2% | 20% | 25% | 25% | 0.433 |
| Renal malignancy <3cm | 21% | 22,4% | 10% | 12,5% | 25% | 0.646 |
| **Bosniak classification** | | | | | | |
| Bosniak I  Bosniak II  Bosniak IIF  Bosniak III  Bosniak IV | 33,9%  39,8%  10%  0,5%  0% | 32,1%  41,2%  9,6%  0,5%  0% | 30,8%  38,5%  7,7%  0%  0% | 43,8% 37,5%  18,8%  0%  0% | 80%  0%  0%  0%  0% | 0.124  0.321  0.566  0.980  n/a |
| **Specialists screening** | | | | | |  |
| (Transplant) surgeon  (Transplant) nephrologist  Anesthesiologist  Social worker  Nurse practitioner  Psychologist/Psychiatrist  Other | 70,6% 82,8%  46,2%  26,7%  41,6%  42,5%  15,8% | 70,1%  81,8%  48,1%  22,5%  42,2%  41,7%  15,5% | 69,2%  76,9%  15,4%  38,5%  15,4%  46,2%  23,1% | 81,3%  100%  50%  56,3%  50%  50%  18,8% | 60%  80%  40%  60%  60%  40%  0% | 0.757  0.285  0.144  0.006  0.182  0.919  0.666 |
| Every donor is discussed in multidisciplinary team | 90,3% | 90,4% | 90% | 93,8% | 75% | 0.731 |
| **Included in multidisciplinary team** | | | | | |  |
| (Transplant) surgeon  (Transplant) nephrologist  Anesthesiologist  Social worker  Nurse practitioner  Psychologist/Psychiatrist  Other | 73,8%  74,2%  38,5%  20,4%  51,6%  38%  21,3% | 72,7%  73,3%  40,1%  13,4%  48,1%  36,9%  19,3% | 69,2%  69,2%  38,5%  61,5%  69,2%  61,5%  46,2% | 93,8%  93,8%  25%  56,3%  75%  31,3%  31,3% | 60%  60%  20%  60%  60%  40%  0% | 0.258  0.264  0.540  0.000  0.104  0.325  0.056 |
| Standard imaging during screening | 100% | 100% | 100% | 100% | 100% | n/a |
| **Type of imaging** | | | | | |  |
| MRI/MRA  CT/CTA  Invasive angiography  Ultrasound  Other | 15,4%  73,3%  4,1%  46,6%  5,9% | 17,6%  70,6%  2,7%  45,5%  5,3% | 7,7%  76,9%  15,4%  46,2%  7,7% | 0%  100%  6,3%  56,3%  12,5% | 0%  80%  20%  60%  0% | 0.162  0.081  0.035  0.787  0.626 |
| Radioisotope renography | 65,4% | 67,7% | 60% | 43,8% | 75% | 0.263 |
| **Functional screening** | | | | | |  |
| MAG-3 scan  DTPA-scan  DMSA-scan  Other | 35,3%  28,1%  19%  13,6% | 37,4%  24,6%  18,2%  13,9% | 23,1%  53,8%  15,4%  0% | 18,8%  43,8%  37,5%  18,8% | 40%  40%  0%  20% | 0.363  0.053  0.178  0.457 |
| **Surgical techniques nephrectomy** | | | | | |  |
| Open (lumbotomy)  Open (mini-incision)  Laparoscopic transperitoneal  Hand-assisted laparoscopic transperitoneal  Retroperitoneoscopic – no hand-assistance  Hand-assisted retroperitoneoscopic  Robot-assisted laparoscopic transperitoneal  Other | 17,6% 25,8%  32,1%  31,2%  5,4%  15,8%  8,6%  2,3% | 15,5%  26,7%  29,9%  29,9%  5,3%  17,1%  10,2%  1,1% | 23,1%  38,5%  23,1%  38,5%  0%  7,7%  0%  23,1% | 37,5%  12,5%  56,3%  37,5%  12,5%  12,5%  0%  0% | 20%  0%  60%  40%  0%  0%  0%  0% | 0.157  0.222  0.074  0.818  0.459  0.583  0.286  0.000 |

| Median + ranges |  |  |  |  |  |  |
| --- | --- | --- | --- | --- | --- | --- |
|  | **Total (n = 101)** | **Europe**  **(n = 85)** | **America (n = 6)** | **Asia**  **(n = 9)** | **Other  (n = 1 )** | **p-value** |
| **BMI** | | | | | |  |
| Overweight (25 – 30)  Obese (30-35)  Morbidly obese (35 - 40)  Morbidly obese (40 +) | 5 (2-5)  3 (1-5) 2 (1-5)  1 (1-5) | 5 (2-5)  3 (1-5)  2 (1-5)  1 (1-5) | 5 (3-5) 4 (2-5)  2 (1-4)  1 (1-3) | 5 (4-5) 4 (2-5)  2 (1-3)  1 (1-2) | 5  5  5  5 | 0.803  0.391  0.253  0.092 |
| Upper age limit for LKD^1^ | 60  (60-no age limit) | No age limit (no age limit-other) | 60  (no age limit to other) | 70  (60 to no age limit) | 80 | 0.053 |
| Minors as donors | 1 (1-5) | 1 (1-5) | 1.5 (1-4) | 1 (1-3) | 1 | 0.618 |
| Women of childbearing age | 4 (1-5) | 4 (1-5) | 5 (4-5) | 4 (2-5) | 2 | 0.026 |
| Impaired fasting glucose | 2 (1-5) | 2 (1-5) | 3 (2-4) | 3 (1-4) | 2 | 0.037 |
| **Hypertension** | | | | | |  |
| Without agents  If well controlled with 1 agent  If well controlled with 2agents  If well controlled with >2agents | 3 (1-5) 4 (1-5) 2 (1-5) 1 (1-5) | 3 (1-5)  4 (1-5)  2 (1-5)  1 (1-5) | 2 (1-4)  4 (1-5)  3.5 (1-4)  2 (1-4) | 4 (1-5)  4 (3-5)  3 (1-4)  2 (1-2) | 3  3  2  2 | 0.249  0.598  0.962  0.318 |
| Arterial or venous anatomy^2^ | 1 (-1-3) | 1 (1-3) | 1.5 (1-3) | 2 (1-3) | 1 | 0.801 |
| **Acceptation number of renal arteries** | | | | | |  |
| 1 renal artery  2 renal arteries  3 renal arteries  4 renal arteries  >4 renal arteries | 5 (3-5)  5 (2-5)  3 (1-5)  2 (1-5)  1 (1-5) | 5 (3-5) 5 (2-5)  3 (1-5)  2 (1-5) 1 (1-5) | 5 (5-5)  5 (4-5)  3 (1-5)  1.5 (1-5)  1 (1-4) | 5 (4-5)  5 (3-5)  3 (1-4)  1 (1-4)  1 (1-3) | 5  5  4  2  2 | 0.530  0.921  0.833  0.934  0.292 |
| **Acceptation number of renal veins** | | | | | |  |
| 1 renal vein  2 renal veins  3 renal veins  4 renal veins  >4 renal veins | 5 (3-5) 5 (1-5)  4 (1-5)  2 (1-5)  2 (1-5) | 5 (3-5)  5 (1-5)  4 (1-5)  2 (1-5)  2 (1-5) | 5 (4-5)  5 (4-5)  3.5 (1-5)  2 (1-3)  1.5 (1-3) | 5 (4-5)  5 (4-5)  3 (1-5)  2 (1-4)  1 (1-4) | 5  5  5  5  5 | 0.454  0.810  0.161  0.357  0.385 |
| **Kidney stones** | | | | | |  |
| Kidney with stones for donation | 3 (1-5) | 3 (1-5) | 4 (2-5) | 4 (2-5) | 2 | 0.389 |
| Stone(s) in contralateral kidney | 2 (1-5) | 2 (1-5) | 2 (1-4) | 2 (1-4) | 2 | 0.770 |
| **Preferred technique(s) nephrectomy** | | | | | |  |
| Open lumbotomy  Open (mini-incision)  Laparoscopic transperitoneal  Hand-assisted laparoscopic transperitoneal  Retroperitoneoscopic, no hand-assistance  Hand-assisted retroperitoneoscopic  Robot-assisted laparoscopic transperitoneal  Other | 6.9%  19.8%  36.6%  31.7%  5.9%  19.8%  4.0%  3.0% | 5.9%  20%  32.9%  31.8%  4.7%  21.2%  4.7%  2.4% | -  33.3%  50%  33.3%  -  -  -  16.7% | 22.2%  11.1%  66.7%  22.2%  22.2%  22.2%  -  - | -  -  -  100%  -  -  -  - | 0.269  0.713  0.169  0.469  0.176  0.601  0.853  0.229 |
| **Percentage of refusal potential LKD** | | | | | |  |
| Percentage of refusal for potential LKD ^4^ | 20-30% (0 – 60%) | 10-20% (0 – 60%) | 20-30% (0-50%) | 20-30% (0-30%) | 0-10% | 0.677 |
| **Deviation from center policy** | | | | | |  |
| Do you sometimes deviate from your center policy | 23.7% | 23.4% | 16.7% | 33.3% | 0% | 0.815 |
| Weight  Blood pressure  Older age  Younger age  Women of childbearing age  Impaired fasting glucose  Vascular multiplicity | 77.3%  36.4%  31.8%  4.5%  13.6%  36.4%  27.3% | 72.2%  27.8%  16.7%  5.6%  11.1%  33.3%  27.8% | 100%  100%  100%  0%  0%  100%  100% | 100%  66.67%  100%  0%  33.33%  33.3%  0% | -  -  -  -  -  -  - | 0.553  0.293  0.007  0.979  0.494  0.819  0.600 |
