# Supplementary material for: Attitudes among transplant professionals regarding shifting paradigms in eligibility criteria for live kidney donation
Source: PLoS One. 2017 Jul 21;12(7):e0181846. doi: 10.1371/journal.pone.0181846 (PMC5521829; doi:10.1371/journal.pone.0181846)
Supplement: S4 Table — (DOCX) [file pone.0181846.s004.docx]

**S4. Table. Analyzed by center volume (number of annual live kidney donations)**

| Mean (SD) |  | **Number of annual live kidney donations** | | | |  |
| --- | --- | --- | --- | --- | --- | --- |
|  | **Europe (n= 187)** | **0 – 25 (n = 83)** | **26 – 50 (n = 39)** | **51 – 100 (n= 32)** | **>100 (n = 13)** | **P-value Overall** |
| **Does your center accept donors with the following BMI-categories?** | | | | | | |
| Overweight | 99,4% | 98,8% | 100% | 100% | 100% | 0.797 |
| Obesity | 69,5% | 54,2% | 79,5% | 84,4% | 100% | < 0.001 |
| Morbid Obesity | 15% | 6% | 12,8% | 25% | 53,8% | < 0.001 |
| Morbid Obesity (Class II) | 4,2% | 1,2% | 0% | 9,1% | 23,1% | 0.001 |
| **Does your center accept minors (<18 years) as donors?** | | | | | | |
|  | 3,6% | 2,4% | 7,7% | 3,1% | 0% | 0.435 |
| **Does your center accept women of childbearing age as donors?** | | | | | | |
|  | 82% | 74,7% | 92,3% | 81,2% | 100% | 0.034 |
| **Does your center accept donors with impaired fasting glucose?** | | | | | | |
|  | 41,5% | 35,8% | 57,1% | 40% | 38,5% | 0.196 |
| **Does your center accept donors with hypertension?** | | | | | | |
| No  If controlled 1 agent  If controlled 2 agents  If controlled with > 2 agents  Yes | 10,1%  47,2%  35,2%  1,3%  6,3% | 0%  61,7%  21%  0%  3,7% | 2,9%  51,4%  40%  2,9%  2,9% | 0%  23,3%  50%  0%  13,3% | 7,7%  0%  76,9%  7,7%  15,4% | < 0.001 |
| **Does your center have an upper age limit for live kidney donors?** | | | | | |  |
| Yes Yes, max 60 Yes, max 65 Yes, max 70 Yes, max 75 Yes, max 80 No age limit | 0,6%  1,9%  8,2%  10,1% 6,9%  5% 67,3% | 1,2%  3,7%  14,8%  16%  8,6%  3,7%  51,9% | 0%  0%  2,9%  2,9%  5,7%  11,4%  77,1% | 0%  0%  0%  3,3%  6,7%  0%  90% | 0%  0%  0%  7,7%  0%  7,7%  84,6% | 0.025 |
| **Does your center accept donors with more than 1 renal artery?** | | | | | | |
| No  Yes, max 2 arteries  Yes, max 3 arteries  Yes, max 4 arteries  Yes, no maximum | 8,2%  40,3%  20,8%  2,5%  28,3% | 16,0%  51,9%  17,3%  1,2%  13,6 | 0%  51,4%  17,1%  8,6%  22,9% | 0%  10%  33,3%  0%  56,7% | 0%  7,7%  23,1%  0%  69,2% | <0.001 |
| **Does your center accept donors with more than 1 renal vein?** | | | | | | |
| No  Yes, max 2 veins  Yes, max 3 veins  Yes, max 4 veins  Yes, no maximum | 10,7%  35,8%  18,2%  0,6%  34,6% | 19,8%  45,7%  13,6%  0%  21,0% | 0%  40,0%  25,7%  0%  34,3% | 3,3%  13,3%  23,3%  3,3  56,7% | 0%  15,4%  15,4%  0%  69,2% | <0.001 |
| **Does your center accept donors with kidney stones?** | | | | | | |
| No  Yes, but only if the remaining kidney is free  Yes | 26,9%  53,8%  19,2% | 40,5%  43,0%  16,5% | 14,3  65,7%  20% | 13,8%  65,5%  20,7% | 7,7%  61,5%  30,8% | 0.014 |
| **Does your center accept donors with one or more kidney stones in the contralateral kidney?** | | | | | | |
|  | 12,2% | 7,6% | 20% | 6,9% | 30,8% | 0.037 |
| **Does our center accept kidneys with a renal malignancy smaller than 3 cm?** | | | | | | |
|  | 22,4% | 13,9% | 31,4% | 24,1% | 46,2% | 0.027 |
| **Does your center accept donors with more renal cysts?** | | | | | | |
| Yes, max Bosniak I  Yes, max Bosniak II  Yes, max Bosniak IIF  Yes, max Bosniak III  Yes, max Bosniak IV | 32,1%  41,2%  9,6%  0,5%  0% | 57,8%  42,2%  7,2%  1,2%  0% | 28,2%  59,0%  10,3%  0%  0% | 59,4%  43,8%  12,5%  0%  0% | 46,2%  76,9%  30,8%  0%  0% | 0,014  0.057  0.086  0.797  - |
| **Which specialist(s) does a donor meet during regular screening in your center?** | | | | | | |
| (Transplant) surgeon  (Transplant) nephrologist  Anesthesiologist  Social worker  Nurse practitioner  Psychologist/Psychiatrist  Other | 70,1%  81,8%  48,1%  22,5%  42,2%  41,7%  15,5% | 79,5%  91,6%  56,6%  18,1%  30,1%  66,3%  22,9% | 74,4%  89,7%  51,3%  23,1%  53,8%  35,9%  15,4% | 71,9%  90,6%  37,5%  31,2%  65,6%  21,9%  6,2% | 100%  100%  84,6%  61,5%  92,3%  15,4%  15,4% | 0.185  0.703  0.033  0.007  0.000  0.000  0.198 |
| **Is every donor discussed in a multidisciplinary team?** | | | | | | |
|  | 90,4% | 93,7% | 82,9% | 89,7% | 92,3% | 0.343 |
| **Which specialist(s) are part of the multidisciplinary team of your center?** | | | | | |  |
| (Transplant) surgeon  (Transplant) nephrologist  Anesthesiologist  Social worker  Nurse practitioner  Psychologist/Psychiatrist  Other | 72,7%  73,3%  40,1%  13,4%  48,1%  36,9%  19,3% | 85,5%  86,7%  53,0%  15,7%  45,8%  62,7%  26,5% | 74,4%  74,4%  43,6%  15,4%  51,3%  30,8%  20,5% | 75,0%  75,0%  15,6%  9,4%  62,5%  9,4%  15,6% | 92,3%  92,3%  69,2%  23,1%  92,3%  15,4%  7,7% | 0.250  0.186  0.001  0.683  0.012  0.000  0.338 |
| **Does your center perform standard pre-operative imaging during the screening of donors?** | | | | | | |
|  | 100% | 100% | 100% | 100% | 100% | - |
| **What modalities of pre-operative imaging are used in your center?** | | | | | |  |
| MRI/MRA  CT/CTA  Invasive angiography  Ultrasound  Other | 17,6%  70,6%  2,7%  45,5%  5,3% | 21,7%  80,7%  3,6%  63,9%  8,4% | 17,9%  76,9%  2,6%  43,6%  5,1% | 18,8%  71,9%  3,1%  37,5%  3,1% | 15,4%  92,3%  0%  23,1%  0% | 0.929  0.454  0.910  0.005  0.527 |
| **Do you perform standard radioisotope renography as part of the live donor screening process?** | | | | | | |
|  | 67,7% | 84,8% | 68,6% | 42,9% | 15,4% | 0.000 |
| **What kind of functional screening do the donors in your center undergo?** | | | | | | |
| MAG-3 scan  DTPA-scan  DMSA-scan  Other | 37,4%  24,6%  18,2%  13,9% | 37.3%  38,6%  19,3%  9,6% | 41,0%  28,2%  23,1%  15,4% | 40,6%  9,4%  18,8%  31,2% | 76,9%  0%  23,1%  15,4% | 0.063  0.002  0.951  0.042 |
| **What kind of surgical techniques are practiced in your center?** | | | | | | |
| Open (lumbotomy)  Open (mini-incision)  Laparoscopic transperitoneal  HALS  Retroperitoneoscopic – no hand-assistance  HARP  Robot-assisted laparoscopic transperitoneal  Other | 15,5%  26,7%  29,9%  29,9%  5,3%  17,1%  10,2%  1,1% | 21,7%  30,1%  32,5%  24,1%  8,4%  3,6%  7,2%  2,4% | 17,9%  38,5%  35,9%  28,2%  2,6%  30,8%  5,1%  0% | 12,5%  25,0%  21,9%  56,2%  3,1%  25,0%  0%  0% | 0%  15,4%  61,5%  53,8%  7,7%  69,2%  84,6%  0% | 0.226  0.386  0.084  0.003  0.527  0.000  0,000  0.562 |

| Median + ranges | **Europe**  **(n = 85)** | **0 – 25 (n = 29)** | **26 – 50 (n = 21)** | **51 – 100**  **(n = 17)** | **> 100 (n = 7)** | **p-value** |
| --- | --- | --- | --- | --- | --- | --- |
| **How likely is it that you would personally perform a live donor nephrectomy in a donor who is:** | | | | | |  |
| Overweight (25 – 30)  Obese (30-35)  Morbidly obese (35 - 40)  Morbidly obese (40 +) | 5 (2-5)  3 (1-5)  2 (1-5)  1 (1-5) | 4 (2-5)  3 (1-4)  1 (1-4)  1 (1-3) | 5 (3-5)  4 (1-5)  1 (1-4)  1 (1-3) | 5 (4-5)  4 (2-5)  2 (1-5)  1 (1-5) | 5 (4-5)  5 (4-5)  4 (2-4)  3 (1-3) | 0.012  0.007  0.000  0.000 |
| Upper age limit for LKD | No age limit (no age limit-other) | 65  (no age limit-other) | No age limit (no age limit-other) | No age limit (no age limit-other) | No age limit (70 – no age limit) | 0.026 |
| Minors as donors | 1 (1-5) | 1 (1-4) | 1 (1-5) | 1 (1-4) | 1 (1-2) | 0.786 |
| Women of childbearing age | 4 (1-5) | 3 (1-5) | 3 (1-5) | 4 (1-5) | 5 (4-5) | 0.116 |
| Impaired fasting glucose | 2 (1-5) | 2 (1-4) | 2 (1-4) | 2 (1-5) | 2 (1-4) | 0.341 |
| **How likely is it that you would personally accept a donor with hypertension with the following conditions:** | | | | | |  |
| Without agents  If well controlled with 1 agent  If well controlled with 2agents  If well controlled with >2agents | 3 (1-5)  4 (1-5)  2 (1-5)  1 (1-5) | 3 (1-5)  4 (2-5)  2 (1-4)  1 (1-3) | 3 (1-5)  4 (2-5)  2 (1-4)  1 (1-3) | 3 (1-5)  4 (1-5)  4 (1-5)  2 (1-4) | 4 (1-5)  5 (4-5)  4 (4-5)  2 (1-5) | 0.453  0.075  0.000  0.004 |
| **What is in your opinion more important, the arterial or the venous anatomy?** | | | | | | |
|  | Artery | Artery | Vein | Artery | Artery | 0.069 |
| **How likely is it that you would personally accept a donor with the following number of renal arteries?** | | | | | | |
| 1 renal artery  2 renal arteries  3 renal arteries  4 renal arteries  >4 renal arteries | 5 (3-5) 5 (2-5)  3 (1-5)  2 (1-5) 1 (1-5) | 5 (4-5)  4 (2-5)  2 (1-5)  1 (1-4)  1 (1-4) | 5  5 (2-5)  3 (1-5)  1 (1-4)  1 (1-3) | 5  5 (4-5)  4 (2-5)  2 (1-5)  1 (1-5) | 5  5  4 (3-5)  3 (2-4)  2 (1-2) | 0.666  0.008  0.026  0.123  0.004 |
| **How likely is it that you would personally accept a donor with the following number of renal veins?** | | | | | | |
| 1 renal vein  2 renal veins  3 renal veins  4 renal veins  >4 renal veins | 5 (3-5)  5 (1-5)  4 (1-5)  2 (1-5)  2 (1-5) | 5 (4-5)  5 (2-5)  4 (1-5)  2 (1-5)  2 (1-5) | 5  5 (1-5)  4 (1-5)  2 (1-5)  1 (1-5) | 5 (4-5)  5 (4-5)  4 (1-5)  2 (1-5)  1 (1-5) | 5  5  4 (3-5)  2 (2-4)  2 (1-2) | 0.684  0.596  0.532  0.142  0.011 |
| **How likely is it that you would personally accept a kidney with stones for donation?** | | | | | |  |
| Kidney with stones for donation | 3 (1-5) | 3 (1-5) | 4 (1-5) | 4 (2-5) | 4 (3-5) | 0.239 |
| Stone(s) in contralateral kidney | 2 (1-5) | 2 (1-3) | 2 (1-5) | 2 (1-4) | 2 (2-3) | 0.173 |
| **Which technique(s) do you preferably use for live donor nephrectomy?** | | | | | | |
| Open lumbotomy  Open (mini-incision)  Laparoscopic transperitoneal  HALS  Retroperitoneoscopic, no hand-assistance  HARP  Robot-assisted laparoscopic transperitoneal  Other | 5.9%  20%  32.9%  31.8%  4.7%  21.2%  4.7%  2.4% | 10,3%  27,6%  41,4%  24,1%  6,9%  0%  6,9%  6,9% | 4,8%  23,8%  28,6%  33,3%  0%  38,1%  4,8%  0% | 0%  17,6%  23,5%  58,8%  5,9%  23,5%  0%  0% | 0%  0%  85,7%  28,6%  0%  85,7%  14,3%  0% | 0.428  0.431  0.026  0.117  0.587  0.000  0.533  0.363 |
| **What is your percentage of refusal for potential live kidney donors?** | | | | | | |
|  | 10-20% (0% – 60%) | 30-40% (0%-other) | 20-30% (0%-other) | 0-10% (0%-other) | 0-10% | 0.026 |
| **Do you sometimes deviate from your center policy?** | | | | | | |
|  | 23.4% | 27,6% | 33,3% | 11,8% | 0% | 0.178 |
| **Regarding which patient characteristic do you deviate?** | | | | | | |
| Weight  Blood pressure  Older age  Younger age  Women of childbearing age  Impaired fasting glucose  Vascular multiplicity | 72.2%  27.8%  16.7%  5.6%  11.1%  33.3%  27.8% | 20,7%  6,9%  10,3%  3,4%  3,4%  6,9%  3,4% | 23,8%  14,3%  0%  0%  4,8%  14,3%  9,5% | 11,8%  0%  0%  0%  0%  0%  5,9% | 0%  0%  0%  0%  0%  0%  0% | 0.449  0.304  0.183  0.666  0.785  0.304  0.724 |
